# Recurrent Pneumonia With Tuberculosis and *Candida* Co-infection Diagnosed by Metagenomic Next-Generation Sequencing: A Case Report and Literature Review

**DOI:** 10.3389/fmed.2022.755308

**Published:** 2022-04-08

**Authors:** Ning Ma, Mei Chen, Jingyi Ding, Fang Wang, Jingbo Jin, Sitong Fan, Jiajia Chen

**Affiliations:** ^1^Department of Infectious Diseases, Beilun District People’s Hospital, Ningbo, China; ^2^State Key Laboratory for Diagnosis and Treatment of Infectious Diseases, The First Affiliated Hospital, Zhejiang University School of Medicine, Hangzhou, China; ^3^Department of Hospital-Acquired Infection Control, Beilun District People’s Hospital, Ningbo, China

**Keywords:** tuberculosis, *Candida* pneumonia, case report, literature review, mNGS

## Abstract

An 82-year-old male patient was hospitalized in the Respiratory Department for “repeated cough and shortness of breath for 10 years, recurrence worsened for 1 month.” Later, he was transferred for further diagnosis and treatment, to the Infectious Disease Department for further hospitalization. Previously, the patient had repeatedly undergone tuberculosis-related examinations including bronchoscopy examinations. However, no evidence of *Mycobacterium tuberculosis* (*MTB*) infection was found. Early anti-infection treatments failed. Due to repeated symptoms, we performed bronchoscopy again and sent alveolar lavage fluid for the metagenomic next-generation sequencing (mNGS) test. Subsequently, *MTB* and *Candida albicans* were detected by mNGS. After antituberculosis and antifungal treatments, the symptoms were significantly relieved, and the chest CT showed resolution of the lung lesions. Therefore, we successfully diagnosed and treated a case of recurrent pneumonia with tuberculosis and *Candida* co-infection diagnosed by mNGS.

## Introduction

Tuberculosis is one of the most important infectious diseases monitored in China, which seriously affects human health. *Mycobacterium tuberculosis* (*MTB*) can invade multiple organs, but tuberculosis is the most common disease they cause. In 2019, approximately 10 million people (range: 8.9–11 million) suffered from tuberculosis (1). Its worldwide prevalence has had a huge impact on the healthcare system both in economic and health terms, which has prompted the World Health Organization (WHO) to include it among the top-priority infectious diseases (2). This study analyzes the clinical data of a patient who suffered from recurrent pneumonia with tuberculosis and *Candida* co-infection, admitted to our department. A review of the available literature on the clinical application of metagenomic next-generation sequencing (mNGS) has also been performed.

## Case Presentation

**FIGURE 1:**
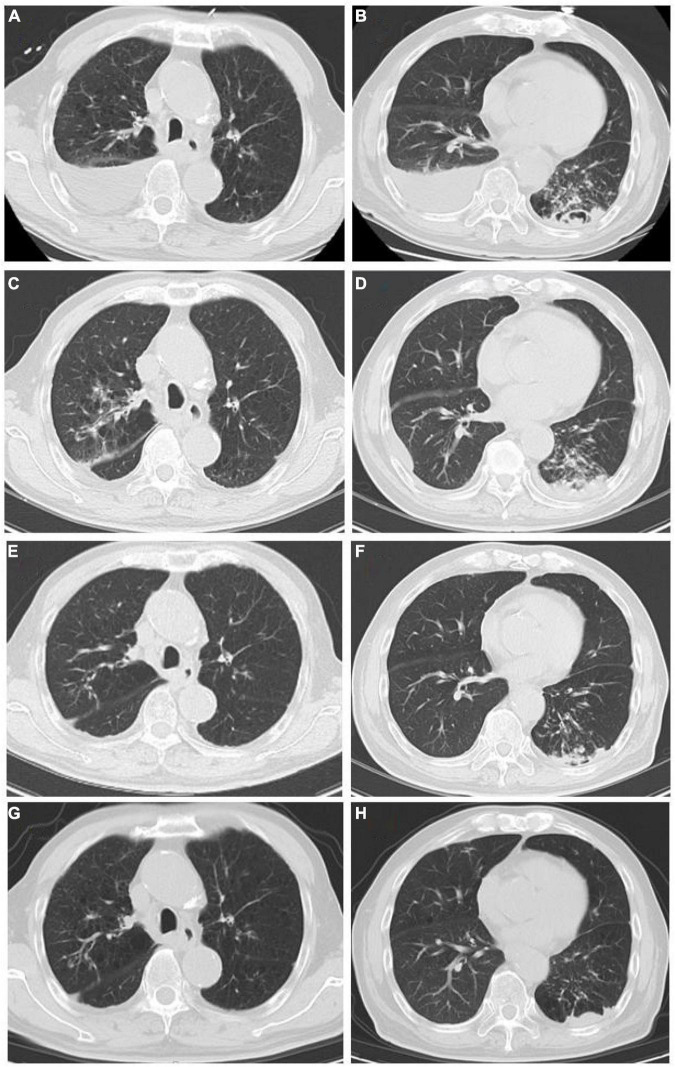
**(A,B)** Chest CT scan at the first admission (June 8). **(A)** Both lungs had increased field permeability; the density of the fluid in the right pleural cavity was affected, and emphysema was considered; the right pleural effusion was accompanied by local atelectasis. **(B)** Bilateral pleural fluid density shadows, multiple patchy high-density shadows in the left lower lung, and a local cavity formation led to suspected bilateral pleural effusion and infectious lesion in the left lung. **(C,D)** Chest CT scan after conventional antibacterial treatments (June 21): Infectious lesions in both lungs increased, left pleural effusion increased, and right pleural effusion decreased as compared to **(A,B)**. **(E,F)** Chest CT scan after the antituberculosis and antifungal treatments (September 2): The scope of infectious lesions in both lungs was the resolution, the left lower lung was more obvious, and the pleural effusions on both sides were significantly reduced as compared to **(C,D)**. **(G,H)** Chest CT at follow-up (December 21): The infectious lesions in both lungs were obviously resolved as compared to **(E,F)**.

An 82-year-old male patient was hospitalized with recurrent cough and shortness of breath for 1 month. Prior to the appearance of these symptoms, he had recurrent cough and shortness of breath for 10 years. He was previously diagnosed with chronic bronchitis. Although his symptoms were slightly relieved after repeated anti-infection (*Piperacillin Sodium/Tazobactam Sodium and Cefoperazone Sodium/Sulbactam Sodium*), anti-asthmatic (*Doxofylline*), and expectorant (*Bromhexine Hydrochloride*) treatments, his cough and shortness of breath were still recurring. At the same time, the patient suffered from weight loss and repeated low-grade fever. A detailed physical examination revealed moist rales in the left lung and diminished respiration in the right lung. Thus, given his poor immunity, it was necessary to consider the infection of pathogens such as *MTB* and fungi. However, repeated examinations showed no pathogenic evidence. The chest CT scan after admission is depicted in Figure 1. Bilateral bronchial disease, bilateral pleural effusion, and lung infection in the right upper lung and left lower lung can be observed on the images. Part of the examination results on admission is presented in Table 1. Due to a large amount of pleural effusion, ultrasound-guided thoracentesis was performed to clarify the nature of the pleural effusion and the adjuvant treatment needed. Meanwhile, the pleural fluid routine, biochemical test, examination of exfoliated cells, fluid culture, and fluid acid-fast bacilli stain were performed, the results of the pleural effusion analysis are listed in Table 2. More than 500 pcs/μl white blood cells were found in the pleural effusion, and the Rivalta test was positive, and exudative pleural effusion needed to be considered. Since the lymphocyte proportion was relatively high, and the purified protein derivative (PPD) was positive, tuberculous pleurisy could not be excluded. However, the Xpert test and γ-interferon release test (T-spot) were negative. In summary, evidence for *MTB* infection was still insufficient. Except for the carbohydrate antigen 125, the tumor markers of the patient were within the normal reference range, and thus, the possibility of tumor involvement was low. However, after the application of conventional antibacterial treatment for 14 days, the patient’s symptoms, signs, and reexamination indicators (Table 1) did not significantly improve. Therefore, bronchoscopy, sputum culture, and fungal examination were performed again. The alveolar lavage fluid was sent for mNGS. Additionally, multiple *MTB* culture identification examinations were performed. The mNGS results revealed the presence of *MTB* (3 reads) and fungi (1,761 reads). The results are displayed in Table 3. *Candida* was isolated in the sputum culture.

**TABLE 1:** Laboratory examination results during the diagnosis and treatment of patients.

Item (unit)	On admission	Ordinary antibacterial treatment for 14 days	Antituberculosis and antifungal treatment for 14 days	Latest follow–up (antituberculosis treatment for half one year)
High-sensitivity C-reactive protein (mg/L)	50.90	99.60	20.20	< 0.5
White blood cell count (×109/L)	3.90	3.50	3.40	-
Neutrophil count (×109/L)	2.56	2.45	2.11	-
Lymphocyte count (×109/L)	0.69	0.49	0.75	-
Albumin (g/L)	31.60	29.3	29.10	35.7
Procalcitonin (ng/ml)	0.05	0.076	0.05	-
Erythrocyte sedimentation rate analysis(mm/h)	78	-	-	26
**Sputum culture**	**Candida**	-	***Mycobacterium tuberculosis***	-
Acid-fast bacilli stain in sputum	Negative	Negative	Negative	Negative
1,3-β-D-glucan detection	Negative	-	-	-
Carbohydrate antigen 125 (U/ml)	129.70	-	-	-
Carbohydrate antigen 19–9 (U/ml)	3.72	-	-	-
Alpha fetoprotein (ng/ml)	1.48	-	-	-
Carcinoembryonic antigen (ng/ml)	1.74	-	-	-

*-, absence of performance.*

**TABLE 2:** Results of the patient’s pleural fluid examinations on admission.

Item	Result
Color	Yellow
Transparency	Turbid
**Rivalta test**	**3+**
Microscopic examination of red blood cell count (pcs/μl)	12,773
**Microscopic examination of white blood cell count (pcs/μ l)**	**2,701**
Neutrophil classification (%)	8
**Lymphocyte classification (%)**	**80**
Percentage of microscopically examined monocytes (%)	12
Total protein (g/L)	11.9
Glucose (mmol/L)	1.33
Lactate dehydrogenase (U/L)	249
Adenosine deaminase (U/L)	13
Pleural fluid culture	Negative
Acid-fast bacilli in pleural fluid	Negative

*Bold values emphasize that these results suggest that pleural effusion may be caused by tuberculosis.*

**TABLE 3:** Patient’s next-generation sequencing (NGS) analysis results (https://www.ncbi.nlm.nih.gov/sra/PRJNA773989).

Genus	Species				
Name	NGS reads^a^	Relative abundance^b^	Name	NGS reads	Relative abundance
Candida	1,758	88.28%	*Candida albicans*	1,607	86.70%
*Mycobacterium tuberculosis complex*	3^c^	0.00	-	-	-

*^a^The NGS reads of the microorganism detected at the genus/species level in rigid alignment.*

*^b^The proportion of the microorganism in all the microorganisms detected.*

*^c^We analyzed them with the BLAST NCBI tool and found that they could be matched to the MTB genome in the NCBI library.*

At present, the diagnosis of *Candida* infection was clear, but the reads of nucleotide sequences of *MTB* were less. The diagnosis of tuberculosis was still in doubt. However, based on the patient’s chest CT scan and pleural effusion tests, *MTB* infection could not be ruled out. Also considering the adverse effects of missing diagnosis and treatment of tuberculosis on the prognosis of patients, we diagnosed the patient with *MTB* and *Candida* co-infection. Then, considering the patient’s advanced age and poor physical condition, we implemented triple antituberculosis (Isoniazid: 300 mg one time a day, Rifampin: 450 mg one time a day, and Levofloxacin: 500 mg one time a day) and antifungal (Fluconazole: 400 mg one time a day) treatments after informed consent was received from the patient. Subsequently, the patient’s laboratory results had improved (Table 1), and Fluconazole was discontinued after 2 weeks. Later, *MTB* was isolated from the patient’s sputum culture (performed on June 14) and alveolar lavage fluid culture (performed on June 15). Hence, the results were reconfirmed. Fortunately, this patient had good adherence, and we followed him up for half a year. Transaminase had been slightly elevated briefly in antituberculosis treatment, and the transaminase was within the normal range after the addition of hepatoprotective drugs. In the subsequent follow-up, we learned that his cough, shortness of breath, and other symptoms were considerably relieved. The lung lesions and pleural effusion were obviously resolved (Figure 1). At present, the drug is orderly reduced to *Isoniazid* and *Rifampin.*

## Materials and Methods

### Sample Processing and DNA Extraction

Notably, a 1.5–3 ml *BALF* sample from the patient was collected according to standard procedures and inactivated in a water bath at 65°C for 30 min; 1.5-ml microcentrifuge tube with 0.6 ml sample and 250 μl 0.5-mm glass bead (weight: 1 g, the concentration was 1,176 mg/ml) were attached to a horizontal platform on a vortex mixer using FastPrep-24™ 5G Instrument and agitated vigorously at 2,800–3,200 rpm for 30 min. Then, 7.2 μl lysozyme (RT410-TA, TIANGEN BIOTECH, Beijing, China) was added for wall-breaking reaction. Of note, 0.3 ml sample was separated into a new 1.5-ml microcentrifuge tube, and DNA was extracted using the TIANamp Micro DNA Kit (DP316, TIANGEN BIOTECH, Beijing, China) according to the manufacturer’s recommendation; concentration was measured by Qubit dsDNA HS Assay Kit 3.0 Fluorometer; samples were taken according to the measured concentration; and the nucleic acid fragment was performed using the nucleic acid enzyme digestion reaction kit produced by Huada Biotechnology (Wuhan) Co., Ltd. (3).

### Construction of DNA Libraries and Sequencing

The PMseq™ High-throughput Detection Kit for infectious pathogens produced by Huada Biotechnology (Wuhan) Co., Ltd. (i.e., combined probe-anchored polymerase sequencing) was used to prepare the DNA libraries. DNA fragmentation, end-repair, adapter-ligation, and unbiased PCR amplification were performed strictly in accordance with the instructions. Agilent 2,100 was used for quality control of the DNA libraries. Quality qualified libraries were pooled, and DNA nanoball was made and sequenced by the BGISEQ-50/MGISEQ-2000 platform (4).

### Bioinformatic Analysis

High-quality sequencing data were generated by removing low-quality reads, followed by computational subtraction of human host sequences mapped to the human reference genome (hg19) using Burrows-Wheeler Alignment (5). The remaining data by removal of low-complexity reads were classified by simultaneously aligning to the pathogen metagenomics database. The classification reference databases were downloaded from NCBI.^1^ RefSeq contains 4,945 whole-genome sequences of viral taxa, 6,350 bacterial genomes or scaffolds, 1,064 fungi related to human infection, and 234 parasites associated with human diseases. We used single-end sequencing, the selected read length was 50 bp, the number of total reads obtained was 50,900,630, and Q30 reached 95.34%.

A positive mNGS was given to bacterial or viral cases when the number of reads mapping to a microbe was 10-fold greater than that of any other microbes, and when the coverage rate for a fungus (species level) was 5-fold greater than that of another fungus, a positive mNGS was given for the fungus. When mNGS detected at least one sequence of the *MTB complex* at the genus level, a positive mNGS was given for *MTB.*

The sputum culture of the patient on admission was *Candida* which we considered as contamination, but his condition had not improved after 14 days of ordinary anti-infective treatment. Referring to the mNGS results, we started antituberculosis and antifungal treatments; later, the sputum culture for *MTB* was positive (performed on June 15), which medium was BD BACTEC™MGIT™ 960 Supplement Kit, which again supported our diagnosis.

## Discussion

Tuberculosis is a disease to be conquered worldwide. Once the diagnosis is clear, 80% of patients can be cured or relieved; at present, the main difficulties are the difficulty of diagnosis and the treatment of multidrug-resistant tuberculosis (6, 7). In this study, a case of recurrent pneumonia with tuberculosis and *Candida* co-infection is reported. The patient had repeatedly suffered from cough and shortness of breath for many years. Multiple bronchoscopies, Xpert, T-spot, and sputum tests for acid-fast bacilli and sputum culture did not detect evidence of *MTB* infection. The efficacy of anti-infection treatment was not effective; therefore, common pathogen infections, tumors, etc., could not explain the patient’s disease characteristics. In such cases, special bacterial infections need to be considered. Nevertheless, the complex infection by *Candida* and *MTB* was detected by the mNGS test. Considering the patient’s age, underlying diseases, and poor immunity, *Candida* and *MTB* were determined to be the infection-causative agents reconfirmed by the results of his sputum culture, excluding the possibility of bacteria colonization and contamination. Finally, after adjustments in the treatment plan and the implementation of antituberculosis and antifungal therapy, the patient’s general condition and reexamination indicators improved. Chest CT reexamination showed slight attenuation in the lung lesions (Figure 1).

Tuberculosis is a chronic contagious disease that seriously endangers human health. The increase in morbidity and mortality from tuberculosis is a consequence of the complex disease process caused by the pathogen *MTB*, which brings a huge burden to society and individuals (8). *MTB* infectious occurs in the lung tissue, trachea, bronchi, and pleura. It can be diagnosed based on the patient’s epidemiological history, clinical symptoms and signs, chest imaging, and laboratory examinations (including bacteriological examination, molecular biological examination, pathological examination, immunological examination and culture, tuberculin skin tests, and tuberculosis antibody tests), bronchoscopy, and other examination methods and tests. The new γ-interferon release test has high sensitivity and specificity. It is not affected by bacillus Calmette-Guérin (BCG) vaccination and body immunity (9). A meta-analysis compared the accuracy of Gene Xpert Assay, Microscopic Observation of Drug Sensitivity (MODS), and WHO 2007 algorithm in the diagnosis of tuberculosis in smear-negative patients and found that the sensitivity rates were 67, 73, and 61%, respectively (10). Based on the different inspection methods and results, cases can be classified as confirmed, clinically diagnosed, or suspected. Despite the progress in diagnostic techniques, a considerable number of clinically diagnosed tuberculosis cases but not bacteriologically confirmed were reported to the WHO. The WHO’s 2020 target for ending tuberculosis aims at the reduction of the incidence of tuberculosis by 20% and the number of deaths from tuberculosis by 35% as compared to 2015 figures (11). Conventional treatment methods cannot meet the requirements of rapid clinical diagnosis. Therefore, new methods need to be developed in China for more effective diagnosis.

With technology advancement, NGS technology, which is high-throughput and can thus perform large-scale gene analysis, has been widely applied in various fields (12). NGS can directly and non-specifically determine all nucleic acid fragments and can obtain the genomic information of all microorganisms in the samples, substantially reducing missed detection (13). mNGS obtains the nucleotide sequence in the sample by means of the NGS platform and further maps it with the genome sequence of each species, so as to know the type and proportion of microorganisms in the sample. Current literature reports that mNGS has been successfully applied to a variety of specimen types such as cerebrospinal fluid, respiratory secretions, and blood (14, 15). Successful diagnosis of rare and difficult-to-diagnose diseases has been gradually achieved over recent years. For example, NGS can reportedly guide the diagnosis and treatment of Mendelian susceptibility to mycobacterial disease (MSMD), *Listeria monocytogenes* infection, aseptic meningitis related to varicella-zoster virus infection, and fungal infection (16–19). A cohort study found that NGS had the highest sensitivity (58.8%) in the diagnosis of tuberculous meningitis as compared to those of Xpert (38.2%) and conventional methods (29.4%) (20). Currently, NGS research on tuberculosis is used mainly for epidemiological investigation, diagnosis, gene polymorphism, and drug resistance detection (13).

A large number of studies have confirmed that NGS can quickly detect multiple genes, facilitating the diagnosis of various pathogens. However, we cannot directly identify them as pathogenic bacteria. For example, if a variety of bacteria and fungi are detected, they must be judged as colonizing bacteria, contaminating bacteria, or pathogenic bacteria. Candidiasis tends to occur in immunocompromised subjects. The prevalence rate is (2.1–21.0)/100,000, and the fatality rate is 40–60%. Its clinical manifestations are diverse, and it is thus difficult to diagnose. *Candida albicans*, smooth *Candida*, and *Candida tropicalis* are the most common pathogens, among which *Candida albicans* accounts for 65–70% (21). Clinically, several methods such as mannan antigen/antibody detection, NGS, 1-3-β-D-glucan test, *Candida* score, and colonization index can be used to assist in determining colonization and infection. But, *Candida* colonization is an important prerequisite for the occurrence of invasive candidiasis. For this reason, it is necessary to combine clinical manifestations and laboratory examinations to make a conclusion to guide the treatment (22, 23). Compared with the huge amount of medical costs, targeted medication can significantly reduce the cost of treatment through defining the etiology using mNGS.

## Conclusion

In case of ineffective therapy of patients with lung infection by conventional anti-infective treatments and persistent lesions, the possibility of infection with *MTB*, fungi, viruses, and specific flora species is to be considered. In cases of suspected *MTB* infection with no evidence of infection obtained by repeated traditional testing methods, novel examination approaches such as mNGS are worthy of consideration to assist in the detection process, which may contribute to facilitating the diagnosis of unspecified infections.

## Data Availability Statement

The datasets presented in this study can be found in online repositories. The names of the repository/repositories and accession number(s) can be found in the article/supplementary material.

## Ethics Statement

The studies involving human participants were reviewed and approved by the Ethics Committee of Beilun District People’s Hospital of Ningbo. The patients/participants provided their written informed consent to participate in this study.

## Author Contributions

NM, MC, and JD wrote the manuscript. JJ and SF carried out the design and corrections of the chart. FW reviewed the manuscript. JC designed the study and reviewed the manuscript. All authors contributed to the article and approved the submitted version.

## Conflict of Interest

The authors declare that the research was conducted in the absence of any commercial or financial relationships that could be construed as a potential conflict of interest.

## Publisher’s Note

All claims expressed in this article are solely those of the authors and do not necessarily represent those of their affiliated organizations, or those of the publisher, the editors and the reviewers. Any product that may be evaluated in this article, or claim that may be made by its manufacturer, is not guaranteed or endorsed by the publisher.

## Footnotes

1
http://ftp.ncbi.nlm.nih.gov/genomes/
